# Supplementary material for: MEME-LaB: motif analysis in clusters
Source: Bioinformatics. 2013 May 14;29(13):1696–7. doi: 10.1093/bioinformatics/btt248 (PMC3694638; doi:10.1093/bioinformatics/btt248)
Supplement: Supplementary Data [file supp_btt248_Supplementary_Data.zip › Supplementary_Data/Results_Files/which_gene_ids/cluster13_motif1.html]

Cluster 13 Motif 1


Cluster 13 Motif 1 is found in the following promoters

| Gene ID | Strand | Position\* |
| --- | --- | --- |

|  |  |  |
| --- | --- | --- |
| AT1G01420 | + | 442/500 |
| AT1G01510 | - | 86/500 |
| AT1G01630 | - | 464/500 |
| AT1G02910 | + | 24/500 |
| AT1G04400 | - | 222/500 |
| AT1G04530 | - | 460/500 |
| AT1G04950 | + | 65/500 |
| AT1G05830 | + | 67/500 |
| AT1G05900 | - | 199/500 |
| AT1G06790 | + | 127/500 |
| AT1G07010 | + | 425/500 |
| AT1G07230 | - | 406/500 |
| AT1G07330 | + | 142/500 |
| AT1G08540 | + | 359/500 |
| AT1G09830 | - | 32/500 |
| AT1G09850 | + | 470/500 |
| AT1G10200 | - | 211/500 |
| AT1G10210 | + | 219/500 |
| AT1G12020 | + | 441/500 |
| AT1G12410 | - | 97/500 |
| AT1G12800 | - | 386/500 |
| AT1G13730 | - | 407/500 |
| AT1G14030 | + | 307/500 |
| AT1G14380 | - | 489/500 |
| AT1G14410 | - | 357/500 |
| AT1G14610 | + | 180/500 |
| AT1G14790 | + | 457/500 |
| AT1G15080 | + | 57/500 |
| AT1G15720 | + | 452/500 |
| AT1G15970 | + | 415/500 |
| AT1G17230 | - | 483/500 |
| AT1G18170 | + | 260/500 |
| AT1G18610 | - | 386/500 |
| AT1G18660 | - | 109/500 |
| AT1G19870 | + | 49/500 |
| AT1G19920 | + | 420/500 |
| AT1G20550 | + | 159/500 |
| AT1G20816 | + | 159/500 |
| AT1G20950 | + | 399/500 |
| AT1G21480 | - | 143/500 |
| AT1G23180 | + | 323/500 |
| AT1G23360 | + | 2/500 |
| AT1G25375 | + | 335/500 |
| AT1G26190 | + | 438/500 |
| AT1G27200 | + | 184/500 |
| AT1G27440 | + | 401/500 |
| AT1G27530 | + | 58/500 |
| AT1G27690 | + | 155/500 |
| AT1G28340 | + | 285/500 |
| AT1G30500 | - | 60/500 |
| AT1G32070 | + | 153/500 |
| AT1G33400 | - | 317/500 |
| AT1G33990 | + | 406/500 |
| AT1G34065 | - | 146/500 |
| AT1G34640 | - | 278/500 |
| AT1G35260 | + | 179/500 |
| AT1G35460 | + | 236/500 |
| AT1G36390 | - | 234/500 |
| AT1G43580 | + | 299/500 |
| AT1G48030 | - | 64/500 |
| AT1G49890 | + | 169/500 |
| AT1G49990 | - | 385/500 |
| AT1G50910 | + | 298/500 |
| AT1G51730 | - | 449/500 |
| AT1G52420 | + | 261/500 |
| AT1G53280 | - | 289/500 |
| AT1G54220 | + | 119/500 |
| AT1G54820 | - | 435/500 |
| AT1G55000 | + | 456/500 |
| AT1G55900 | - | 242/500 |
| AT1G56000 | - | 166/500 |
| AT1G56050 | - | 120/500 |
| AT1G56190 | + | 273/500 |
| AT1G56460 | + | 296/500 |
| AT1G57790 | + | 335/500 |
| AT1G60230 | + | 12/500 |
| AT1G61010 | - | 460/500 |
| AT1G61960 | - | 185/500 |
| AT1G62520 | - | 228/500 |
| AT1G62830 | - | 130/500 |
| AT1G63850 | + | 223/500 |
| AT1G63970 | - | 37/500 |
| AT1G65320 | - | 127/500 |
| AT1G66250 | + | 132/500 |
| AT1G67280 | - | 474/500 |
| AT1G67480 | - | 449/500 |
| AT1G68780 | - | 76/500 |
| AT1G69390 | - | 371/500 |
| AT1G70730 | - | 286/500 |
| AT1G72190 | - | 321/500 |
| AT1G72310 | + | 321/500 |
| AT1G72500 | + | 486/500 |
| AT1G72550 | - | 97/500 |
| AT1G73110 | + | 278/500 |
| AT1G73530 | + | 489/500 |
| AT1G73655 | + | 70/500 |
| AT1G73940 | + | 305/500 |
| AT1G73960 | - | 141/500 |
| AT1G74940 | + | 289/500 |
| AT1G75280 | - | 1/500 |
| AT1G77800 | - | 39/500 |
| AT1G78040 | - | 375/500 |
| AT1G78880 | + | 139/500 |
| AT1G80170 | - | 133/500 |
| AT1G80600 | - | 193/500 |
| AT2G01630 | + | 294/500 |
| AT2G01680 | + | 408/500 |
| AT2G02680 | - | 181/500 |
| AT2G03505 | + | 311/500 |
| AT2G03810 | + | 229/500 |
| AT2G04230 | - | 471/500 |
| AT2G05790 | + | 399/500 |
| AT2G06010 | - | 348/500 |
| AT2G14890 | + | 143/500 |
| AT2G15280 | + | 335/500 |
| AT2G16750 | - | 223/500 |
| AT2G19860 | + | 54/500 |
| AT2G20230 | - | 75/500 |
| AT2G20370 | - | 486/500 |
| AT2G20890 | + | 161/500 |
| AT2G21010 | + | 10/500 |
| AT2G21970 | - | 480/500 |
| AT2G23300 | + | 326/500 |
| AT2G23460 | - | 437/500 |
| AT2G24390 | + | 366/500 |
| AT2G25480 | - | 49/500 |
| AT2G26340 | - | 260/500 |
| AT2G26640 | + | 450/500 |
| AT2G26930 | + | 470/500 |
| AT2G27130 | + | 470/500 |
| AT2G27230 | + | 359/500 |
| AT2G28290 | - | 144/500 |
| AT2G30620 | + | 404/500 |
| AT2G31040 | - | 364/500 |
| AT2G31660 | + | 258/500 |
| AT2G31670 | - | 272/500 |
| AT2G32520 | + | 147/500 |
| AT2G32640 | + | 354/500 |
| AT2G33340 | + | 249/500 |
| AT2G35450 | + | 107/500 |
| AT2G35470 | - | 180/500 |
| AT2G35840 | + | 236/500 |
| AT2G36990 | + | 417/500 |
| AT2G38270 | + | 356/500 |
| AT2G38330 | + | 358/500 |
| AT2G38650 | + | 476/500 |
| AT2G40520 | - | 216/500 |
| AT2G41080 | - | 180/500 |
| AT2G41090 | + | 404/500 |
| AT2G41500 | - | 445/500 |
| AT2G41530 | - | 156/500 |
| AT2G41630 | - | 274/500 |
| AT2G41950 | - | 323/500 |
| AT2G43745 | + | 444/500 |
| AT2G44300 | - | 48/500 |
| AT2G44760 | + | 253/500 |
| AT2G45530 | - | 3/500 |
| AT2G46420 | - | 483/500 |
| AT2G46915 | + | 182/500 |
| AT2G47070 | - | 480/500 |
| AT2G47790 | + | 74/500 |
| AT2G48070 | - | 345/500 |
| AT3G01510 | - | 353/500 |
| AT3G02110 | - | 402/500 |
| AT3G02720 | - | 459/500 |
| AT3G03040 | + | 381/500 |
| AT3G03250 | - | 3/500 |
| AT3G04310 | - | 283/500 |
| AT3G05650 | + | 286/500 |
| AT3G06950 | - | 380/500 |
| AT3G07410 | - | 184/500 |
| AT3G09250 | + | 106/500 |
| AT3G10160 | + | 459/500 |
| AT3G10350 | + | 435/500 |
| AT3G10630 | - | 483/500 |
| AT3G11070 | - | 326/500 |
| AT3G11280 | - | 343/500 |
| AT3G11830 | + | 270/500 |
| AT3G12110 | + | 94/500 |
| AT3G12210 | - | 31/500 |
| AT3G13040 | + | 155/500 |
| AT3G13810 | - | 457/500 |
| AT3G14220 | - | 93/500 |
| AT3G14410 | + | 305/500 |
| AT3G14690 | + | 193/500 |
| AT3G14860 | - | 294/500 |
| AT3G15390 | + | 135/500 |
| AT3G17210 | + | 294/500 |
| AT3G17350 | + | 363/500 |
| AT3G17840 | + | 311/500 |
| AT3G17850 | + | 239/500 |
| AT3G18160 | + | 451/500 |
| AT3G18390 | + | 221/500 |
| AT3G18420 | - | 283/500 |
| AT3G19170 | - | 393/500 |
| AT3G19450 | - | 64/500 |
| AT3G19480 | - | 371/500 |
| AT3G19590 | + | 9/500 |
| AT3G19720 | + | 363/500 |
| AT3G19810 | - | 188/500 |
| AT3G20120 | - | 482/500 |
| AT3G20350 | + | 200/500 |
| AT3G21200 | - | 139/500 |
| AT3G21740 | + | 290/500 |
| AT3G23110 | + | 151/500 |
| AT3G23400 | + | 233/500 |
| AT3G23690 | - | 476/500 |
| AT3G23710 | + | 89/500 |
| AT3G23830 | + | 111/500 |
| AT3G23940 | + | 184/500 |
| AT3G24030 | + | 255/500 |
| AT3G24190 | - | 351/500 |
| AT3G24520 | + | 333/500 |
| AT3G24590 | - | 452/500 |
| AT3G25430 | + | 300/500 |
| AT3G25710 | + | 83/500 |
| AT3G26750 | - | 205/500 |
| AT3G27570 | + | 117/500 |
| AT3G27750 | + | 229/500 |
| AT3G27840 | - | 409/500 |
| AT3G28910 | - | 315/500 |
| AT3G28920 | + | 296/500 |
| AT3G29090 | + | 243/500 |
| AT3G43220 | + | 437/500 |
| AT3G45290 | - | 37/500 |
| AT3G46450 | - | 247/500 |
| AT3G46550 | + | 151/500 |
| AT3G47510 | + | 456/500 |
| AT3G48040 | + | 418/500 |
| AT3G48110 | - | 410/500 |
| AT3G48530 | + | 33/500 |
| AT3G48750 | + | 450/500 |
| AT3G49140 | + | 155/500 |
| AT3G52050 | + | 372/500 |
| AT3G52155 | + | 319/500 |
| AT3G53760 | + | 425/500 |
| AT3G54360 | + | 279/500 |
| AT3G54390 | + | 28/500 |
| AT3G54440 | + | 254/500 |
| AT3G54470 | - | 187/500 |
| AT3G54910 | + | 148/500 |
| AT3G55040 | - | 184/500 |
| AT3G55080 | + | 74/500 |
| AT3G56480 | + | 319/500 |
| AT3G57930 | + | 301/500 |
| AT3G57990 | + | 140/500 |
| AT3G58140 | + | 135/500 |
| AT3G58590 | - | 384/500 |
| AT3G59490 | + | 155/500 |
| AT3G60240 | - | 483/500 |
| AT3G60350 | + | 378/500 |
| AT3G60440 | - | 245/500 |
| AT3G61700 | - | 60/500 |
| AT3G61750 | - | 90/500 |
| AT3G62200 | + | 100/500 |
| AT3G62300 | - | 242/500 |
| AT3G62970 | + | 15/500 |
| AT3G63120 | - | 488/500 |
| AT3G66658 | + | 134/500 |
| AT4G00650 | - | 478/500 |
| AT4G00710 | - | 456/500 |
| AT4G01570 | - | 4/500 |
| AT4G04950 | + | 158/500 |
| AT4G09550 | - | 439/500 |
| AT4G09640 | + | 197/500 |
| AT4G10180 | - | 130/500 |
| AT4G10800 | - | 267/500 |
| AT4G11410 | - | 102/500 |
| AT4G12390 | + | 436/500 |
| AT4G12670 | + | 484/500 |
| AT4G13360 | - | 319/500 |
| AT4G13430 | - | 295/500 |
| AT4G13590 | - | 290/500 |
| AT4G14570 | - | 417/500 |
| AT4G14620 | + | 225/500 |
| AT4G14713 | - | 185/500 |
| AT4G15570 | + | 116/500 |
| AT4G16120 | + | 463/500 |
| AT4G16390 | - | 449/500 |
| AT4G16442 | + | 163/500 |
| AT4G17050 | - | 359/500 |
| AT4G17616 | + | 172/500 |
| AT4G18370 | + | 91/500 |
| AT4G20090 | + | 294/500 |
| AT4G20960 | - | 272/500 |
| AT4G21445 | + | 381/500 |
| AT4G21705 | - | 335/500 |
| AT4G22670 | - | 190/500 |
| AT4G25570 | + | 291/500 |
| AT4G25780 | - | 332/500 |
| AT4G25830 | + | 200/500 |
| AT4G25860 | - | 92/500 |
| AT4G26430 | + | 400/500 |
| AT4G26500 | - | 350/500 |
| AT4G26900 | - | 181/500 |
| AT4G28030 | - | 282/500 |
| AT4G28640 | + | 369/500 |
| AT4G28740 | - | 215/500 |
| AT4G29070 | - | 391/500 |
| AT4G29080 | + | 413/500 |
| AT4G29680 | + | 266/500 |
| AT4G30110 | - | 468/500 |
| AT4G30310 | - | 282/500 |
| AT4G30580 | - | 113/500 |
| AT4G30610 | - | 81/500 |
| AT4G30845 | + | 451/500 |
| AT4G30993 | + | 244/500 |
| AT4G31350 | + | 410/500 |
| AT4G31390 | + | 319/500 |
| AT4G31450 | - | 192/500 |
| AT4G31850 | + | 55/500 |
| AT4G31990 | - | 293/500 |
| AT4G32260 | + | 194/500 |
| AT4G32420 | + | 324/500 |
| AT4G32520 | - | 267/500 |
| AT4G33080 | - | 314/500 |
| AT4G33380 | + | 272/500 |
| AT4G33630 | - | 327/500 |
| AT4G33680 | + | 173/500 |
| AT4G34030 | - | 396/500 |
| AT4G36240 | + | 144/500 |
| AT4G36390 | - | 385/500 |
| AT4G36860 | - | 312/500 |
| AT4G37000 | + | 477/500 |
| AT4G37040 | + | 219/500 |
| AT4G37470 | + | 63/500 |
| AT4G37590 | + | 303/500 |
| AT4G38020 | + | 317/500 |
| AT4G39190 | + | 423/500 |
| AT4G39900 | + | 41/500 |
| AT5G01020 | - | 314/500 |
| AT5G01220 | - | 293/500 |
| AT5G01230 | + | 60/500 |
| AT5G01470 | - | 128/500 |
| AT5G01590 | + | 188/500 |
| AT5G02130 | + | 339/500 |
| AT5G02890 | + | 94/500 |
| AT5G03420 | + | 227/500 |
| AT5G03550 | + | 275/500 |
| AT5G04110 | - | 66/500 |
| AT5G04740 | - | 65/500 |
| AT5G05480 | + | 94/500 |
| AT5G05830 | + | 266/500 |
| AT5G06060 | - | 164/500 |
| AT5G06440 | - | 230/500 |
| AT5G08390 | + | 441/500 |
| AT5G08430 | - | 479/500 |
| AT5G08570 | + | 99/500 |
| AT5G09770 | + | 420/500 |
| AT5G10060 | - | 384/500 |
| AT5G11450 | + | 168/500 |
| AT5G11580 | - | 185/500 |
| AT5G11590 | + | 349/500 |
| AT5G11910 | - | 182/500 |
| AT5G12260 | + | 383/500 |
| AT5G12320 | + | 107/500 |
| AT5G13310 | + | 350/500 |
| AT5G13410 | - | 450/500 |
| AT5G13570 | + | 370/500 |
| AT5G13690 | - | 351/500 |
| AT5G13700 | + | 187/500 |
| AT5G14590 | - | 306/500 |
| AT5G15770 | - | 213/500 |
| AT5G15980 | - | 473/500 |
| AT5G16390 | - | 250/500 |
| AT5G16550 | + | 401/500 |
| AT5G16610 | + | 476/500 |
| AT5G16870 | - | 442/500 |
| AT5G17410 | + | 213/500 |
| AT5G17710 | + | 484/500 |
| AT5G18590 | - | 286/500 |
| AT5G19540 | - | 410/500 |
| AT5G19620 | - | 438/500 |
| AT5G20280 | - | 418/500 |
| AT5G21060 | - | 333/500 |
| AT5G22700 | - | 231/500 |
| AT5G22840 | + | 126/500 |
| AT5G24165 | - | 298/500 |
| AT5G24300 | + | 431/500 |
| AT5G24650 | + | 488/500 |
| AT5G24690 | - | 241/500 |
| AT5G25480 | + | 287/500 |
| AT5G27730 | + | 406/500 |
| AT5G27820 | + | 164/500 |
| AT5G28840 | - | 278/500 |
| AT5G41210 | + | 342/500 |
| AT5G41260 | + | 394/500 |
| AT5G41761 | + | 254/500 |
| AT5G42450 | - | 462/500 |
| AT5G43270 | - | 332/500 |
| AT5G44660 | + | 358/500 |
| AT5G45390 | - | 471/500 |
| AT5G45490 | - | 466/500 |
| AT5G46160 | - | 345/500 |
| AT5G46390 | - | 348/500 |
| AT5G46570 | + | 199/500 |
| AT5G47040 | + | 409/500 |
| AT5G47210 | - | 97/500 |
| AT5G47780 | - | 300/500 |
| AT5G47840 | + | 274/500 |
| AT5G48610 | + | 48/500 |
| AT5G49740 | - | 463/500 |
| AT5G50110 | + | 448/500 |
| AT5G50250 | + | 138/500 |
| AT5G51080 | - | 167/500 |
| AT5G51540 | + | 113/500 |
| AT5G51560 | - | 461/500 |
| AT5G52100 | + | 363/500 |
| AT5G53900 | - | 37/500 |
| AT5G55630 | + | 455/500 |
| AT5G55910 | + | 395/500 |
| AT5G57100 | - | 316/500 |
| AT5G57180 | - | 468/500 |
| AT5G57210 | + | 253/500 |
| AT5G57850 | + | 32/500 |
| AT5G57940 | - | 471/500 |
| AT5G58003 | + | 106/500 |
| AT5G58870 | + | 475/500 |
| AT5G58920 | - | 38/500 |
| AT5G59010 | + | 317/500 |
| AT5G60600 | - | 18/500 |
| AT5G61030 | - | 105/500 |
| AT5G61130 | - | 249/500 |
| AT5G61930 | - | 218/500 |
| AT5G62100 | - | 400/500 |
| AT5G62520 | + | 228/500 |
| AT5G62650 | + | 439/500 |
| AT5G62790 | + | 109/500 |
| AT5G63420 | - | 137/500 |
| AT5G63440 | + | 68/500 |
| AT5G63830 | + | 205/500 |
| AT5G64000 | - | 318/500 |
| AT5G64340 | - | 336/500 |
| AT5G65020 | + | 178/500 |
| AT5G65683 | + | 468/500 |
| AT5G66055 | + | 13/500 |
| AT5G66180 | + | 302/500 |
| AT5G67130 | - | 273/500 |
| AT5G67360 | + | 467/500 |

**\*** Overlapping sequences are merged to avoid redundancy. As a result some sequences in the final set may be longer than the specified maximum.

Distribution

+

-

Position values represent number of bases from the 5 prime end of the sequence provided to MEME and the length of that sequence. It is these figures expressed as proportions that are used for statistical analysis.

Probability value of comparison of these values to a linear distribution, p = 0.0024

The motif is found 238 times on the + stand and 201 times on the - strand.

Probability value that it is evenly distributed between the two strands, p = 0.043

Move the mouse pointer over the gene ids to highlight the corresponding marker
